# 4,4,6-Trimethyl-1-(3-methyl­phen­yl)-3,4-dihydro­pyrimidine-2(1*H*)-thione

**DOI:** 10.1107/S1600536810004708

**Published:** 2010-02-13

**Authors:** Aamer Saeed, Rasheed Ahmad Khera, Masood Parvez

**Affiliations:** a Department of Chemistry, Quaid-i-Azam University, Islamabad 45320, Pakistan; b Department of Chemistry, The University of Calgary, 2500 University Drive NW, Calgary, Alberta, Canada T2N 1N4

## Abstract

The asymmetric unit of the title compound, C_14_H_18_N_2_S, contains two independent and conformationally similar mol­ecules, which form cyclic dimers *via* inter­molecular hydrogen bonds of the type N—H⋯S [graph set *R* ^2^_2_(8)]. The structure is iso­morphous with that of one of the polymorphs of 4,4,6-tri­methyl-1-phenyl-3,4-dihydro­pyrimidine-2(1*H*)-thione [Yam­in *et al.* (2005 ▶). *Acta Cryst.* E**61**, o55–o57].

## Related literature

For the biological activity of pyrimidine-2-thio­nes, see: Alam *et al.* (2005 ▶); Sriram *et al.* (2006 ▶); Swamy *et al.* (2005 ▶). For related structures, see: Yamin *et al.* (2005 ▶); Ismail *et al.* (2007 ▶). For graph-set analysis, see Etter *et al.* (1990 ▶).
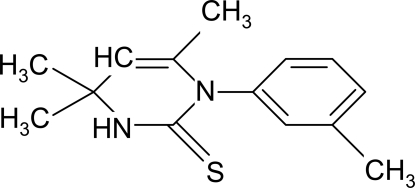


## Experimental

### 

#### Crystal data

C_14_H_18_N_2_S
*M* *_r_* = 246.36
Orthorhombic, 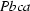

*a* = 10.5904 (3) Å
*b* = 16.9189 (5) Å
*c* = 30.5713 (10) Å
*V* = 5477.7 (3) Å^3^
*Z* = 16
Mo *K*α radiation
μ = 0.22 mm^−1^
*T* = 173 K
0.14 × 0.12 × 0.08 mm

#### Data collection

Bruker APEXII CCD diffractometer
Absorption correction: multi-scan (*SORTAV*; Blessing, 1997 ▶) *T*_min_ = 0.970, *T*_max_ = 0.983
19135 measured reflections
4830 independent reflections
3231 reflections with *I* > 2.0σ(*I*)
*R*_int_ = 0.041

#### Refinement

*R*[*F* ^2^ > 2σ(*F* ^2^)] = 0.049
*wR*(*F* ^2^) = 0.115
*S* = 1.04
4830 reflections
315 parameters
H-atom parameters constrained
Δρ_max_ = 0.25 e Å^−3^
Δρ_min_ = −0.23 e Å^−3^

### 

Data collection: *COLLECT* (Hooft, 1998 ▶); cell refinement: *HKL *DENZO** (Otwinowski & Minor, 1997 ▶); data reduction: *SCALEPACK* (Otwinowski & Minor, 1997 ▶); program(s) used to solve structure: *SHELXS97* (Sheldrick, 2008 ▶); program(s) used to refine structure: *SHELXL97* (Sheldrick, 2008 ▶); molecular graphics: *ORTEP-3 for Windows* (Farrugia, 1997 ▶); software used to prepare material for publication: *SHELXTL* (Sheldrick, 2008 ▶).

## Supplementary Material

Crystal structure: contains datablocks Global, I. DOI: 10.1107/S1600536810004708/zs2028sup1.cif

Structure factors: contains datablocks I. DOI: 10.1107/S1600536810004708/zs2028Isup2.hkl

Additional supplementary materials:  crystallographic information; 3D view; checkCIF report

## Figures and Tables

**Table 1:** Hydrogen-bond geometry (Å, °)

*D*—H⋯*A*	*D*—H	H⋯*A*	*D*⋯*A*	*D*—H⋯*A*
N1—H1*N*⋯S2	0.88	2.62	3.419 (2)	151
N3—H3*N*⋯S1	0.88	2.60	3.447 (2)	163

**Fig. 1.**
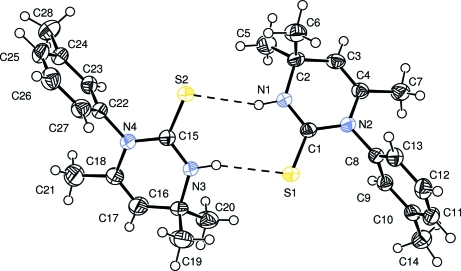
ORTEP-3 (Farrugia, 1997) drawing of the two molecules in the asymmetric unit of (I) with displacement ellipsoids plotted at 50% probability level. Hydrogen bonds have been drawn with dashed lines.

## supplementary crystallographic information

### Comment

The title compound C_14_H_18_N_2_S (I) belongs to a novel and rare class of dihydropyrimidine-2-thiones. Their synthesis has been attracting widespread attention because of their diverse pharmacological properties such as antibacterial (Alam *et al.*, 2005), antitumour (Swamy *et al.*, 2005) and antioxidative activities (Sriram *et al.*, 2006). The crystal structure of a closely related compound, 4,4,6-trimethyl-1-phenyl-3,4-dihydropyrimidine-2(1*H*)-thione (Yamin *et al.*, 2005) and its triclinic polymorph (Ismail *et al.*, 2007) have been reported.

The asymmetric unit of the title compound is composed of two molecules (Fig. 1) exhibiting intermolecular hydrogen bonds of the type N—H···S (Table 1) forming cyclic dimers [graph set R^2^_2_(8) (Etter *et al.*, 1990)]. The two molecules are conformationally similar [e.g. the inter-ring torsion angle C1–N2–C8–C13 (molecule 1), -83.1 (3)° and C15–N4–C22–C27, -85.9 (3)° (molecule 2)] and are related by pseudo two-fold rotational symmetry. The title compound is isomorphous with one of the polymorphs of 4,4,6-trimethyl-1-phenyl-3,4-dihydropyrimidine-2(1*H*)-thione (Yamin *et al.*, 2005). However, in the triclinic polymorph of this related compound (Ismail *et al.*, 2007), which also forms a cyclic dimer, the two molecules have crystallographic inversion symmetry.

### Experimental

The title compound was prepared by the reaction of 3-methylaniline with 4-methylpent-3-en-2-one in the presence of potassium thiocyanate in acetone: details of the synthesis will be reported later. Recrystallization from methanol afforded the title compound as colourless crystals: Anal. calcd. for C_14_H_18_N_2_S: C, 68.25; H, 7.36; N, 11.37; S, 13.01%; found: C, 68.09; H, 7.41; N, 11.51; S, 13.12.

### Refinement

All H-atoms were visible in the difference Fourier maps but were included in the refinements at geometrically idealized positions with distances: N—H = 0.88 Å and C—H = 0.95 and 0.98 Å, with U_iso_ = 1.2*U*_eq_ of the atoms to which they were bonded. The final difference map was free of chemically significant features.

### Crystal data

**Table:**

C_14_H_18_N_2_S	*F*(000) = 2112
*M**_r_* = 246.36	*D*_x_ = 1.195 Mg m^−^^3^
Orthorhombic, *P**b**c**a*	Mo *K*α radiation, λ = 0.71073 Å
Hall symbol: -P 2ac 2ab	Cell parameters from 8634 reflections
*a* = 10.5904 (3) Å	θ = 1.0–25.3°
*b* = 16.9189 (5) Å	µ = 0.22 mm^−^^1^
*c* = 30.5713 (10) Å	*T* = 173 K
*V* = 5477.7 (3) Å^3^	Block, colorless
*Z* = 16	0.14 × 0.12 × 0.08 mm

### Data collection

**Table:**

Bruker APEXII CCD diffractometer	4830 independent reflections
Radiation source: fine-focus sealed tube	3231 reflections with *I* > 2.0σ(*I*)
graphite	*R*_int_ = 0.041
φ & ω scans	θ_max_ = 25.3°, θ_min_ = 2.3°
Absorption correction: multi-scan (*SORTAV*; Blessing, 1997)	*h* = −12→12
*T*_min_ = 0.970, *T*_max_ = 0.983	*k* = −20→20
19135 measured reflections	*l* = −36→36

### Refinement

**Table:**

Refinement on *F*^2^	Primary atom site location: structure-invariant direct methods
Least-squares matrix: full	Secondary atom site location: difference Fourier map
*R*[*F*^2^ > 2σ(*F*^2^)] = 0.049	Hydrogen site location: inferred from neighbouring sites
*wR*(*F*^2^) = 0.115	H-atom parameters constrained
*S* = 1.04	*w* = 1/[σ^2^(*F*_o_^2^) + (0.038*P*)^2^ + 3.05*P*] where *P* = (*F*_o_^2^ + 2*F*_c_^2^)/3
4830 reflections	(Δ/σ)_max_ < 0.001
315 parameters	Δρ_max_ = 0.25 e Å^−^^3^
0 restraints	Δρ_min_ = −0.23 e Å^−^^3^

### Special details

**Table:**

Geometry. All esds (except the esd in the dihedral angle between two l.s. planes) are estimated using the full covariance matrix. The cell esds are taken into account individually in the estimation of esds in distances, angles and torsion angles; correlations between esds in cell parameters are only used when they are defined by crystal symmetry. An approximate (isotropic) treatment of cell esds is used for estimating esds involving l.s. planes.
Refinement. Refinement of *F*^2^ against ALL reflections. The weighted *R*-factor wR and goodness of fit *S* are based on *F*^2^, conventional *R*-factors *R* are based on *F*, with *F* set to zero for negative *F*^2^. The threshold expression of *F*^2^ > σ(*F*^2^) is used only for calculating *R*-factors(gt) etc. and is not relevant to the choice of reflections for refinement. *R*-factors based on *F*^2^ are statistically about twice as large as those based on *F*, and *R*- factors based on ALL data will be even larger.

### Fractional atomic coordinates and isotropic or equivalent isotropic displacement parameters (Å^2^)

**Table:**

	*x*	*y*	*z*	*U*_iso_*/*U*_eq_	
S1	0.85314 (7)	0.07897 (4)	0.325070 (19)	0.03644 (19)	
S2	0.84290 (7)	0.24913 (4)	0.43316 (2)	0.03863 (19)	
N1	0.75860 (19)	0.22309 (11)	0.32616 (6)	0.0343 (5)	
H1N	0.7579	0.2164	0.3547	0.041*	
N2	0.76802 (18)	0.16559 (11)	0.25790 (6)	0.0305 (5)	
N3	0.7806 (2)	0.09853 (11)	0.43444 (6)	0.0376 (5)	
H3N	0.7897	0.1032	0.4060	0.045*	
N4	0.7743 (2)	0.16054 (12)	0.50157 (6)	0.0354 (5)	
C1	0.7907 (2)	0.16081 (13)	0.30203 (7)	0.0285 (6)	
C2	0.7240 (2)	0.30212 (14)	0.30983 (8)	0.0327 (6)	
C3	0.6741 (2)	0.29264 (14)	0.26468 (8)	0.0365 (6)	
H3	0.6220	0.3332	0.2530	0.044*	
C4	0.6990 (2)	0.23009 (14)	0.23982 (8)	0.0325 (6)	
C5	0.6260 (3)	0.33607 (17)	0.34098 (9)	0.0513 (8)	
H5A	0.6052	0.3902	0.3321	0.062*	
H5B	0.6601	0.3365	0.3708	0.062*	
H5C	0.5496	0.3034	0.3401	0.062*	
C6	0.8409 (3)	0.35435 (16)	0.31010 (10)	0.0476 (7)	
H6A	0.8182	0.4077	0.3003	0.057*	
H6B	0.9045	0.3321	0.2903	0.057*	
H6C	0.8753	0.3569	0.3398	0.057*	
C7	0.6571 (3)	0.22143 (16)	0.19344 (8)	0.0440 (7)	
H7A	0.6124	0.2694	0.1843	0.053*	
H7B	0.6003	0.1759	0.1909	0.053*	
H7C	0.7309	0.2133	0.1746	0.053*	
C8	0.8098 (2)	0.10194 (14)	0.23002 (7)	0.0293 (6)	
C9	0.7269 (2)	0.04236 (14)	0.21939 (7)	0.0316 (6)	
H9	0.6433	0.0432	0.2306	0.038*	
C10	0.7653 (2)	−0.01925 (14)	0.19210 (8)	0.0346 (6)	
C11	0.8885 (3)	−0.01932 (15)	0.17714 (8)	0.0380 (6)	
H11	0.9167	−0.0617	0.1592	0.046*	
C12	0.9715 (2)	0.04068 (16)	0.18757 (8)	0.0386 (6)	
H12	1.0554	0.0397	0.1766	0.046*	
C13	0.9320 (2)	0.10269 (15)	0.21418 (7)	0.0339 (6)	
H13	0.9879	0.1447	0.2213	0.041*	
C14	0.6721 (3)	−0.08216 (16)	0.17965 (9)	0.0494 (8)	
H14A	0.7167	−0.1264	0.1658	0.059*	
H14B	0.6104	−0.0602	0.1591	0.059*	
H14C	0.6283	−0.1009	0.2059	0.059*	
C15	0.7974 (2)	0.16412 (14)	0.45766 (8)	0.0325 (6)	
C16	0.7484 (3)	0.01867 (14)	0.45061 (8)	0.0390 (7)	
C17	0.7067 (3)	0.02656 (17)	0.49684 (9)	0.0549 (8)	
H17	0.6667	−0.0176	0.5101	0.066*	
C18	0.7217 (3)	0.09111 (15)	0.52074 (8)	0.0420 (7)	
C19	0.8654 (3)	−0.03424 (16)	0.44798 (10)	0.0569 (8)	
H19A	0.8435	−0.0878	0.4576	0.068*	
H19B	0.9317	−0.0129	0.4670	0.068*	
H19C	0.8959	−0.0359	0.4177	0.068*	
C20	0.6454 (3)	−0.01520 (17)	0.42148 (10)	0.0532 (8)	
H20A	0.6238	−0.0686	0.4314	0.064*	
H20B	0.6756	−0.0174	0.3912	0.064*	
H20C	0.5704	0.0186	0.4230	0.064*	
C21	0.6857 (4)	0.09640 (19)	0.56755 (10)	0.0709 (10)	
H21A	0.6561	0.0447	0.5777	0.085*	
H21B	0.6180	0.1354	0.5710	0.085*	
H21C	0.7591	0.1127	0.5849	0.085*	
C22	0.8055 (2)	0.22713 (14)	0.52939 (8)	0.0331 (6)	
C23	0.7123 (2)	0.27825 (15)	0.54251 (8)	0.0355 (6)	
H23	0.6288	0.2718	0.5317	0.043*	
C24	0.7391 (3)	0.33970 (15)	0.57156 (8)	0.0381 (6)	
C25	0.8616 (3)	0.34748 (16)	0.58649 (8)	0.0425 (7)	
H25	0.8815	0.3887	0.6064	0.051*	
C26	0.9557 (3)	0.29648 (17)	0.57300 (8)	0.0464 (7)	
H26	1.0396	0.3033	0.5833	0.056*	
C27	0.9278 (3)	0.23536 (16)	0.54441 (8)	0.0403 (7)	
H27	0.9918	0.1997	0.5353	0.048*	
C28	0.6363 (3)	0.39498 (17)	0.58604 (10)	0.0541 (8)	
H28A	0.6659	0.4260	0.6111	0.065*	
H28B	0.5617	0.3644	0.5945	0.065*	
H28C	0.6144	0.4306	0.5619	0.065*	

### Atomic displacement parameters (Å^2^)

**Table:**

	*U*^11^	*U*^22^	*U*^33^	*U*^12^	*U*^13^	*U*^23^
S1	0.0510 (4)	0.0257 (3)	0.0326 (3)	0.0049 (3)	−0.0024 (3)	0.0000 (3)
S2	0.0582 (5)	0.0254 (3)	0.0322 (3)	−0.0035 (3)	−0.0002 (3)	0.0002 (3)
N1	0.0497 (13)	0.0252 (11)	0.0281 (10)	0.0039 (10)	0.0008 (10)	−0.0014 (9)
N2	0.0352 (12)	0.0272 (11)	0.0292 (10)	0.0041 (10)	−0.0024 (10)	−0.0019 (9)
N3	0.0615 (15)	0.0240 (11)	0.0274 (11)	−0.0043 (11)	0.0009 (11)	−0.0011 (9)
N4	0.0493 (14)	0.0276 (11)	0.0291 (11)	−0.0034 (11)	0.0026 (10)	−0.0016 (9)
C1	0.0295 (13)	0.0243 (13)	0.0318 (13)	−0.0033 (11)	0.0000 (11)	−0.0012 (10)
C2	0.0375 (15)	0.0245 (13)	0.0362 (13)	0.0026 (12)	−0.0003 (12)	−0.0029 (11)
C3	0.0383 (15)	0.0272 (14)	0.0439 (15)	0.0046 (12)	−0.0074 (13)	0.0029 (12)
C4	0.0326 (14)	0.0304 (14)	0.0345 (14)	0.0000 (12)	−0.0033 (12)	0.0016 (11)
C5	0.0586 (19)	0.0457 (18)	0.0495 (16)	0.0192 (16)	0.0048 (15)	−0.0015 (14)
C6	0.0503 (18)	0.0360 (16)	0.0564 (17)	−0.0044 (14)	−0.0067 (15)	0.0013 (13)
C7	0.0522 (18)	0.0370 (16)	0.0427 (15)	0.0072 (14)	−0.0087 (14)	0.0001 (12)
C8	0.0337 (15)	0.0272 (13)	0.0270 (12)	0.0039 (12)	−0.0030 (11)	0.0003 (10)
C9	0.0310 (14)	0.0335 (14)	0.0304 (13)	0.0018 (12)	0.0000 (11)	0.0007 (11)
C10	0.0437 (16)	0.0299 (14)	0.0300 (13)	0.0001 (13)	−0.0069 (12)	0.0011 (11)
C11	0.0471 (17)	0.0375 (16)	0.0293 (13)	0.0127 (14)	−0.0058 (13)	−0.0053 (12)
C12	0.0360 (15)	0.0475 (17)	0.0324 (14)	0.0066 (14)	0.0006 (12)	−0.0001 (13)
C13	0.0338 (15)	0.0375 (15)	0.0305 (13)	−0.0008 (12)	−0.0011 (12)	−0.0006 (11)
C14	0.061 (2)	0.0392 (16)	0.0475 (16)	−0.0060 (15)	−0.0062 (15)	−0.0059 (13)
C15	0.0375 (15)	0.0285 (14)	0.0316 (13)	0.0011 (12)	−0.0027 (11)	−0.0003 (11)
C16	0.0558 (18)	0.0235 (14)	0.0378 (15)	−0.0044 (13)	0.0016 (14)	0.0008 (11)
C17	0.083 (2)	0.0356 (17)	0.0457 (17)	−0.0191 (17)	0.0134 (16)	0.0015 (13)
C18	0.0555 (18)	0.0337 (15)	0.0369 (14)	−0.0072 (14)	0.0043 (14)	0.0028 (12)
C19	0.067 (2)	0.0353 (17)	0.068 (2)	0.0020 (16)	−0.0107 (17)	0.0072 (15)
C20	0.060 (2)	0.0351 (16)	0.0645 (19)	−0.0108 (15)	−0.0136 (16)	0.0014 (14)
C21	0.114 (3)	0.054 (2)	0.0451 (17)	−0.026 (2)	0.0167 (19)	0.0044 (15)
C22	0.0427 (16)	0.0287 (14)	0.0278 (12)	−0.0003 (13)	0.0009 (12)	−0.0017 (10)
C23	0.0359 (15)	0.0386 (15)	0.0319 (13)	−0.0014 (13)	−0.0031 (12)	−0.0013 (12)
C24	0.0479 (17)	0.0339 (15)	0.0325 (13)	0.0029 (14)	0.0026 (13)	−0.0058 (12)
C25	0.0503 (18)	0.0429 (17)	0.0343 (14)	−0.0076 (15)	−0.0065 (14)	−0.0086 (12)
C26	0.0418 (17)	0.0542 (19)	0.0431 (16)	−0.0059 (16)	−0.0066 (14)	0.0004 (14)
C27	0.0400 (16)	0.0384 (16)	0.0425 (15)	0.0029 (13)	0.0002 (13)	−0.0008 (12)
C28	0.0571 (19)	0.0471 (18)	0.0580 (18)	0.0050 (16)	0.0054 (16)	−0.0155 (15)

### Geometric parameters (Å, °)

**Table:**

S1—C1	1.688 (2)	C11—H11	0.9500
S2—C15	1.692 (2)	C12—C13	1.392 (3)
N1—C1	1.331 (3)	C12—H12	0.9500
N1—C2	1.473 (3)	C13—H13	0.9500
N1—H1N	0.8800	C14—H14A	0.9800
N2—C1	1.373 (3)	C14—H14B	0.9800
N2—C4	1.425 (3)	C14—H14C	0.9800
N2—C8	1.443 (3)	C16—C17	1.487 (4)
N3—C15	1.329 (3)	C16—C20	1.520 (4)
N3—C16	1.478 (3)	C16—C19	1.531 (4)
N3—H3N	0.8800	C17—C18	1.324 (4)
N4—C15	1.366 (3)	C17—H17	0.9500
N4—C18	1.426 (3)	C18—C21	1.484 (4)
N4—C22	1.450 (3)	C19—H19A	0.9800
C2—C3	1.487 (3)	C19—H19B	0.9800
C2—C5	1.521 (3)	C19—H19C	0.9800
C2—C6	1.521 (3)	C20—H20A	0.9800
C3—C4	1.329 (3)	C20—H20B	0.9800
C3—H3	0.9500	C20—H20C	0.9800
C4—C7	1.493 (3)	C21—H21A	0.9800
C5—H5A	0.9800	C21—H21B	0.9800
C5—H5B	0.9800	C21—H21C	0.9800
C5—H5C	0.9800	C22—C23	1.372 (3)
C6—H6A	0.9800	C22—C27	1.381 (3)
C6—H6B	0.9800	C23—C24	1.396 (3)
C6—H6C	0.9800	C23—H23	0.9500
C7—H7A	0.9800	C24—C25	1.382 (4)
C7—H7B	0.9800	C24—C28	1.501 (4)
C7—H7C	0.9800	C25—C26	1.381 (4)
C8—C9	1.376 (3)	C25—H25	0.9500
C8—C13	1.381 (3)	C26—C27	1.386 (4)
C9—C10	1.396 (3)	C26—H26	0.9500
C9—H9	0.9500	C27—H27	0.9500
C10—C11	1.382 (4)	C28—H28A	0.9800
C10—C14	1.501 (3)	C28—H28B	0.9800
C11—C12	1.380 (3)	C28—H28C	0.9800
			
C1—N1—C2	126.47 (19)	C10—C14—H14A	109.5
C1—N1—H1N	116.8	C10—C14—H14B	109.5
C2—N1—H1N	116.8	H14A—C14—H14B	109.5
C1—N2—C4	121.07 (19)	C10—C14—H14C	109.5
C1—N2—C8	118.86 (19)	H14A—C14—H14C	109.5
C4—N2—C8	119.99 (18)	H14B—C14—H14C	109.5
C15—N3—C16	128.0 (2)	N3—C15—N4	117.6 (2)
C15—N3—H3N	116.0	N3—C15—S2	120.77 (18)
C16—N3—H3N	116.0	N4—C15—S2	121.60 (18)
C15—N4—C18	120.7 (2)	N3—C16—C17	107.7 (2)
C15—N4—C22	120.1 (2)	N3—C16—C20	108.3 (2)
C18—N4—C22	119.22 (19)	C17—C16—C20	112.2 (2)
N1—C1—N2	117.0 (2)	N3—C16—C19	109.3 (2)
N1—C1—S1	121.21 (17)	C17—C16—C19	110.0 (2)
N2—C1—S1	121.80 (17)	C20—C16—C19	109.3 (2)
N1—C2—C3	107.76 (19)	C18—C17—C16	124.3 (2)
N1—C2—C5	107.5 (2)	C18—C17—H17	117.9
C3—C2—C5	112.3 (2)	C16—C17—H17	117.9
N1—C2—C6	108.8 (2)	C17—C18—N4	120.0 (2)
C3—C2—C6	110.9 (2)	C17—C18—C21	123.5 (3)
C5—C2—C6	109.4 (2)	N4—C18—C21	116.6 (2)
C4—C3—C2	123.1 (2)	C16—C19—H19A	109.5
C4—C3—H3	118.4	C16—C19—H19B	109.5
C2—C3—H3	118.4	H19A—C19—H19B	109.5
C3—C4—N2	119.3 (2)	C16—C19—H19C	109.5
C3—C4—C7	124.2 (2)	H19A—C19—H19C	109.5
N2—C4—C7	116.5 (2)	H19B—C19—H19C	109.5
C2—C5—H5A	109.5	C16—C20—H20A	109.5
C2—C5—H5B	109.5	C16—C20—H20B	109.5
H5A—C5—H5B	109.5	H20A—C20—H20B	109.5
C2—C5—H5C	109.5	C16—C20—H20C	109.5
H5A—C5—H5C	109.5	H20A—C20—H20C	109.5
H5B—C5—H5C	109.5	H20B—C20—H20C	109.5
C2—C6—H6A	109.5	C18—C21—H21A	109.5
C2—C6—H6B	109.5	C18—C21—H21B	109.5
H6A—C6—H6B	109.5	H21A—C21—H21B	109.5
C2—C6—H6C	109.5	C18—C21—H21C	109.5
H6A—C6—H6C	109.5	H21A—C21—H21C	109.5
H6B—C6—H6C	109.5	H21B—C21—H21C	109.5
C4—C7—H7A	109.5	C23—C22—C27	120.9 (2)
C4—C7—H7B	109.5	C23—C22—N4	119.8 (2)
H7A—C7—H7B	109.5	C27—C22—N4	119.2 (2)
C4—C7—H7C	109.5	C22—C23—C24	120.6 (2)
H7A—C7—H7C	109.5	C22—C23—H23	119.7
H7B—C7—H7C	109.5	C24—C23—H23	119.7
C9—C8—C13	121.5 (2)	C25—C24—C23	118.1 (2)
C9—C8—N2	119.4 (2)	C25—C24—C28	121.6 (2)
C13—C8—N2	119.2 (2)	C23—C24—C28	120.3 (2)
C8—C9—C10	120.1 (2)	C26—C25—C24	121.3 (2)
C8—C9—H9	119.9	C26—C25—H25	119.3
C10—C9—H9	119.9	C24—C25—H25	119.3
C11—C10—C9	118.3 (2)	C25—C26—C27	120.0 (3)
C11—C10—C14	122.4 (2)	C25—C26—H26	120.0
C9—C10—C14	119.3 (2)	C27—C26—H26	120.0
C12—C11—C10	121.6 (2)	C22—C27—C26	119.0 (3)
C12—C11—H11	119.2	C22—C27—H27	120.5
C10—C11—H11	119.2	C26—C27—H27	120.5
C11—C12—C13	119.9 (2)	C24—C28—H28A	109.5
C11—C12—H12	120.1	C24—C28—H28B	109.5
C13—C12—H12	120.1	H28A—C28—H28B	109.5
C8—C13—C12	118.7 (2)	C24—C28—H28C	109.5
C8—C13—H13	120.7	H28A—C28—H28C	109.5
C12—C13—H13	120.7	H28B—C28—H28C	109.5
			
C2—N1—C1—N2	−12.6 (4)	C16—N3—C15—N4	−5.1 (4)
C2—N1—C1—S1	168.88 (18)	C16—N3—C15—S2	176.2 (2)
C4—N2—C1—N1	−7.6 (3)	C18—N4—C15—N3	−6.2 (4)
C8—N2—C1—N1	175.5 (2)	C22—N4—C15—N3	172.5 (2)
C4—N2—C1—S1	170.90 (17)	C18—N4—C15—S2	172.50 (19)
C8—N2—C1—S1	−6.0 (3)	C22—N4—C15—S2	−8.8 (3)
C1—N1—C2—C3	25.6 (3)	C15—N3—C16—C17	13.6 (4)
C1—N1—C2—C5	146.8 (2)	C15—N3—C16—C20	135.1 (3)
C1—N1—C2—C6	−94.7 (3)	C15—N3—C16—C19	−105.9 (3)
N1—C2—C3—C4	−21.0 (3)	N3—C16—C17—C18	−12.5 (4)
C5—C2—C3—C4	−139.2 (3)	C20—C16—C17—C18	−131.6 (3)
C6—C2—C3—C4	98.0 (3)	C19—C16—C17—C18	106.6 (3)
C2—C3—C4—N2	5.3 (4)	C16—C17—C18—N4	3.8 (5)
C2—C3—C4—C7	−175.9 (2)	C16—C17—C18—C21	−176.6 (3)
C1—N2—C4—C3	11.0 (3)	C15—N4—C18—C17	6.8 (4)
C8—N2—C4—C3	−172.1 (2)	C22—N4—C18—C17	−171.9 (3)
C1—N2—C4—C7	−167.8 (2)	C15—N4—C18—C21	−172.8 (3)
C8—N2—C4—C7	9.1 (3)	C22—N4—C18—C21	8.5 (4)
C1—N2—C8—C9	94.8 (3)	C15—N4—C22—C23	100.7 (3)
C4—N2—C8—C9	−82.2 (3)	C18—N4—C22—C23	−80.6 (3)
C1—N2—C8—C13	−85.9 (3)	C15—N4—C22—C27	−83.1 (3)
C4—N2—C8—C13	97.1 (3)	C18—N4—C22—C27	95.6 (3)
C13—C8—C9—C10	0.1 (4)	C27—C22—C23—C24	−0.2 (4)
N2—C8—C9—C10	179.3 (2)	N4—C22—C23—C24	175.8 (2)
C8—C9—C10—C11	1.4 (3)	C22—C23—C24—C25	0.2 (4)
C8—C9—C10—C14	−177.9 (2)	C22—C23—C24—C28	−179.5 (2)
C9—C10—C11—C12	−1.8 (4)	C23—C24—C25—C26	0.4 (4)
C14—C10—C11—C12	177.4 (2)	C28—C24—C25—C26	−179.9 (3)
C10—C11—C12—C13	0.8 (4)	C24—C25—C26—C27	−0.9 (4)
C9—C8—C13—C12	−1.2 (4)	C23—C22—C27—C26	−0.2 (4)
N2—C8—C13—C12	179.5 (2)	N4—C22—C27—C26	−176.4 (2)
C11—C12—C13—C8	0.8 (3)	C25—C26—C27—C22	0.8 (4)

### Hydrogen-bond geometry (Å, °)

**Table:**

*D*—H···*A*	*D*—H	H···*A*	*D*···*A*	*D*—H···*A*
N1—H1N···S2	0.88	2.62	3.419 (2)	151
N3—H3N···S1	0.88	2.60	3.447 (2)	163
